# Evaluation of *Habenaria aitchisonii* Reichb. for antioxidant, anti-inflammatory, and antinociceptive effects with *in vivo* and *in silico* approaches

**DOI:** 10.3389/fchem.2024.1351827

**Published:** 2024-03-19

**Authors:** Saeed Ahmed Asiri, Madeeha Shabnam, Rehman Zafar, Osama M. Alshehri, Mohammed Ali Alshehri, Abdul Sadiq, Mater H. Mahnashi, Muhammad Saeed Jan

**Affiliations:** ^1^ Department of Clinical Laboratory Sciences, Faculty of Applied Medical Sciences, Najran University, Najran, Saudi Arabia; ^2^ Department of Chemistry, Women University, Mardan, Khyber Pakhtunkhwa, Pakistan; ^3^ Akhtar Saeed College of Pharmacy, Rawalpindi, Pakistan; ^4^ Department of Clinical Laboratory Sciences, College of Applied Medical Sciences, Najran University, Najran, Saudi Arabia; ^5^ Department of Pharmacy, Faculty of Biological Sciences, University of Malakand, Chakdara, Khyber Pakhtunkhwa, Pakistan; ^6^ Department of Pharmaceutical Chemistry, College of Pharmacy, Najran University, Najran, Saudi Arabia; ^7^ Department of Pharmacy, Bacha Khan University, Charsadda, Khyber Pakhtunkhwa, Pakistan

**Keywords:** *Habenaria aitchisonii*, COX-2, 5-LOX, antioxidant, anti-inflammatory, antinociception

## Abstract

*Habenaria aitchisonii* Reichb was analyzed in this research, including its chemical composition and its *in vitro* antioxidant, anti-inflammatory, acute oral toxicity, and antinociceptive activity. The chloroform and ethyl acetate fractions were found to be the most powerful based on *in vitro* antioxidant, anti-inflammatory, and analgesic assays. The acute oral toxicity of the crude methanolic extract was determined before *in vivo* studies. The acetic acid and formalin tests were used to measure the antinociceptive effect, and the potential mechanisms involved in antinociception were explored. The carrageenan-induced paw edema test was used to examine the immediate anti-inflammatory effect, and many phlogistic agents were used to determine the specific mechanism. Furthermore, for *ex vivo* activities, the mice were sacrificed, the forebrain was isolated, and the antioxidant levels of glutathione (GSH), superoxide dismutase (SOD), thiobarbituric acid reactive substances (TBARS) and catalase (CAT) were estimated using a UV spectrophotometer. No toxicity was seen at oral dosages up to 3,000 mg/kg. The antinociceptive impact was much higher than the standard drug. Both the inflammatory and neurogenic phases of the formalin experiment revealed an analgesic effect in the chloroform and ethyl acetate fractions. In carrageenan anti-inflammatory assays, the chloroform fraction (Ha.Chf) was the most potent fraction. We further studied the GC-MS of crude plant extract and found a total of 18 compounds. In the anti-inflammatory mechanism, it was observed that the Ha.Chf inhibits the COX-2 as well as 5-LOX pathways. The results exhibited that this species is a good source of phytocomponents like germacrone, which can be employed as a sustainable and natural therapeutic agent, supporting its traditional use in folk medicine for inflammatory conditions and pain.

## Graphical Abstract

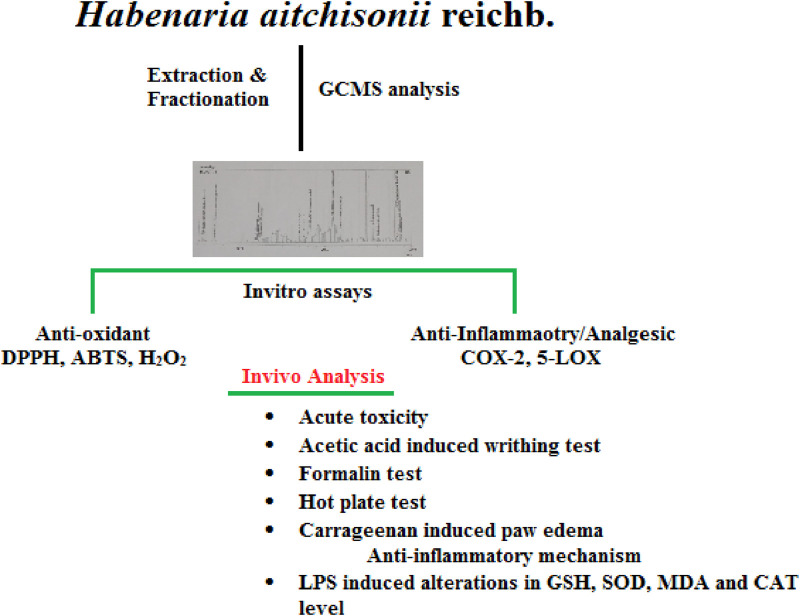

## 1 Introduction

Natural products are demonstrated for various pharmacological and medicinal purposes. Therapeutic potential may be found in medicinal plants due to their different chemical scaffolds (Ibrahim and Gabr, 2019; Nichols et al., 2022). Both their active ingredients and byproducts are employed in the pharmaceutical industry. Infusions, decoctions, mixtures, and teas containing their extracts have been utilized medicinally throughout human history to treat a wide range of ailments accompanied by pain and inflammation (Vickers and Altman, 2001). Various NSAIDs are available for the management of pain and inflammation, but they may produce various side effects such as bleeding, ulcers, difficulty with urination, and seizures (Alam et al., 2020; Javed et al., 2021). Only 15% of the approximately 300,000 species of terrestrial plants described have been systematically studied for their biological capabilities and/or phytochemical profiles (D’Angelo, 2023). A literature survey revealed that herbal-derived compounds have minimum side effects with excellent efficacy and economical status (Houghton, 1995; Abbas et al., 2022). The pain and inflammatory mediators initiate from lipid peroxidation in which arachidonic acid is formed, which further converts to prostaglandins (PGs) and leukotrienes following different pathways with the help of COX-2 and 5-LOX. Similarly, the inhibition of COX-2 and 5-LOX inhibits the formation of PGs and leukotrienes, so the pain and inflammation are relieved and avoided (Martel-Pelletier et al., 2003; Ahsan et al., 2023). In this modern era, many new diseases are leading to increased human mortality (Shah et al., 2014; Jan et al., 2024). To overcome different threats to human health and various side effects of synthetic drugs (Sadiq et al., 2018), researchers have been focused on the development of new medicinal natural products to treat different health-related problems (Zahoor et al., 2018).

Free radicals, also known as reactive oxygen species (ROS), are formed as a result of oxidative stress and have been hypothesized as a possible reason for a variety of pathological illnesses such as cancer, analgesia, atherosclerosis, neurological disorders, diabetes, and inflammation. ROS cause inflammation by promoting cytokine production and activation of enzymes such as lipoxygenases (LOXs) from inflammatory cells. LOX is implicated in the development of various inflammatory disorders (Baiseitova et al., 2023).

All of the 25,000 to 35,000 species of orchids worldwide (Chase et al., 2015) belong to the family Orchidaceae, and *Habenaria* is a widely distributed genus. The Orchidaceae family is eminent in ethnomedicine and has been used in traditional remedies for a variety of ailments in different areas (Misra et al., 2020). Orchids have been employed for thousands of years to treat a variety of conditions, from stomach disorders to arthritis, jaundice, syphilis, acidity, tumors, inflammations, piles, blood dysentery, earaches, malaria, wounds, cholera, eczema, diarrhea, and vermifuge (Hossain, 2011; Ramos et al., 2012). Orchidaceae has a long history of usage as traditional medicine, most often for treating inflammation and pain (Barragán-Zarate et al., 2020). *Dendrobium (*Orchidaceae) was first described as a stimulant for the treatment of pain and inflammation in Chinese pharmacopoeia by approximately 200 B.C. (Gopal et al., 2006). Recently, the aerial part of the folk medicine *Bulbophyllum neilgherrense* has been studied for its potential as an anti-inflammatory and pain reliever (Sadiq et al., 2021). Researchers are currently working on the anti-inflammatory effects of various South African orchid species. It has been observed that the chemical components of Orchidaceae species exhibit numerous powerful biological activities (Mahnashi et al., 2021). *Himantoglossum robertianum* and *Stachys lavandulifolia*, both members of the family Orchidaceae, contain polyphenolic compounds with anti-inflammatory, analgesic, and skin-protective properties (Branine et al., 2019). Similarly, *Cyrtopodium andersonii*, *Bletilla striata*, and *Dendrobium denneanum*, all from the same family, have exhibited promising anti-inflammatory effects (Lin et al., 2013; Parente et al., 2014; Wang and Meng, 2015). Likewise, strong anti-inflammatory and antioxidant effects of orchid family members like *Vanda coerulea* and *Eulophia macrobulbon*, *Trichosanthes dioica*, *Geodorum densiflorum*, *Diospyros blancoi*, *Phragmipedium longifolium*, *Baccaurea ramiflora*, and Da Chuan Xiong Fang have been explored in the literature (Ali et al., 2006; Chen et al., 2013; Waszczak et al., 2015). The literature survey revealed that the pharmacological potential of *H. aitchisonii* Reichb. has not yet been investigated. In the current study, we explored *H. aitchisonii* Reichb. for its analgesic, anti-inflammatory, and antioxidant properties due to its traditional therapeutic use. In addition, we used GC-MS analysis to identify the phytochemicals. We also studied the bioactive compounds to determine their synergistic impact through molecular docking studies.

## 2 Materials and methods

### 2.1 Chemicals and drugs

Sigma-Aldrich was chosen as the local supplier for the chemicals, drugs, and solvents used in this research. Arachidonic acid (CAT No: 152386) and linoleic acid (CAS No: 60-32-2) are fatty acids that are precursors to lipoxygenase (5-LOX) and cyclooxygenase (COX-2), respectively. Glutathione (GSH) (CAS 72-16-6), *N, N,N,N*-tetramethyl-p-phenylenediamine dihydrochloride (TMPD) (CAS 637-01-4), and hematin (CAS No. 15485-92-6) from Sigma-Aldrich serve as cofactors and indicators. Analytical grade solvents were used.

### 2.2 Plant material collection and identification

H. *aitchisonii* Reichb. was collected in the Ayubia region of Galyat, Pakistan, in the months of April–July 2022. The plant material was identified by Professor Muhammad Ibrar, Department of Pharmacognosy at Bacha Khan University, Charsadda. The plant material was deposited under voucher number 03-BKUC/2022 at the herbarium.

### 2.3 Extraction and fractionation

The plant materials (15 Kg) were rinsed in sterile water and shade-dried for 3 weeks. The powder was macerated in methanol (26 L, 80% concentration). After that, Whatman filter paper and muslin cloth were used to remove any remaining debris (Huneif et al., 2022). Finally, 650 g of a dark greenish solid *H. aitchisonii* methanolic extract was obtained. A separating funnel with a closed stopper was carefully filled with the *H. aitchisonii* (Ha.Cr) methanolic extract. Aliquots (500 mL) of water and n-hexane (500 mL) were added to dilute the Ha.Cr. The polarity of the solvents was increased, and the process was repeated. The ensuing solvent fractions attained were 42 g of chloroform, 30 g of ethyl acetate, and 94 g of n-butanol. A final concentration of 140 g was achieved in the aqueous layer.

### 2.4 GC-MS analysis (phytochemistry)

The methanolic extract (Ha.Cr) was analyzed using gas chromatography/mass spectrometry using an Agilent USB-393752 gas chromatograph (Agilent Technologies, USA) fitted with an HHP-5MS 5% phenyl-methylsiloxane tubular column (30 m × 0.25 mm x 0.25 m film thickness; Restek, Bellefonte, PA) (Khan et al., 2022).

### 2.5 *In vitro* anti-inflammatory assays

#### 2.5.1 COX-2 assay

The standard COX-2 anti-inflammatory assay procedure was followed. The COX-2 enzyme solution was made with a concentration range of 300 U/mL. The 10 µL enzyme solution was placed on ice for 5–10 min to activate the enzyme. In addition, 50 μL of a cofactor solution was added to the enzyme solution, which included 0.9 mM GSH, 0.24 mM TMPD, and 1 mM hematin in 0.1 M Tris-HCl buffer at pH 8.0. Then, for 5 min at 25°C, we mixed 60 μL of enzyme solution with 20 μL of the tested samples at varying concentrations (15.625 μg/mL–250 μg/mL). Similarly, 30 mM arachidonic acid (20 μL) was added to start the reaction. Then, the solution was incubated for 4 min. The absorbance was measured at 570 nm after incubation using a UV-visible spectrophotometer (Model 300BB, Thermo Electronic Corporation, England). The COX-2% enzyme inhibition was measured via the absorbance value for a certain time interval. The IC_50_ values were calculated via plotting % enzyme inhibition against various concentrations of tested samples. Celecoxib was used as the reference drug in this assay (Jan et al., 2020).

#### 2.5.2 5-LOX assay

The *H. aitchisonii* crude and subsequent fractions were subjected to a 5-LOX inhibitory assay as per the previously published protocol (Alqahtani et al., 2022). We used various plant samples at concentrations ranging from 15.625 μg/mL to 250 μg/mL. Then, we prepared the enzyme 5-LOX using a 10,000 U/mL solution. Linoleic acid (80 mM) was used as a substrate in this assay. The lipoxygenase enzyme solution (250 μL) was added to different concentrations of the plant sample solutions. 

Then, 0.6 mM of the substrate solution was mixed with the enzyme solution, and the mixture was shaken vigorously before the absorbance was read at 234 nm. Triplicate runs of each experiment were conducted. Zileuton was used as a reference drug in this assay. The following equation was used to calculate the percentage inhibition:

$$\%\text{ Inhibition}=\text{Abs}._{\left(\text{Control}\right)}-\text{Abs}._{\left(\text{Sample}\right)}/\text{Abs}._{\left(\text{Control}\right)}\times 100.$$

### 2.6 *In vitro* antioxidant activities

#### 2.6.1 DPPH assay

A DPPH free radical scavenging assay was carried out for the crude and subsequent fractions, as reported previously (Begum et al., 2019; Mir et al., 2019). Standard dilutions of all samples were created in a range from 250 μg/mL to 15.625 μg/mL. A specific amount of the plant solution (100 µL) was added to the freshly made DPPH (0.004%) solution. All samples were incubated for 15 min to allow the DPPH to scavenge the free radicals. After that, the sample and standard drug were observed using a UV spectrophotometer at a wavelength of 517 nm. Ascorbic acid was employed as a standard.

#### 2.6.2 ABTS assay

The ABTS free radical inhibitory potential procedure was carried out as reported in our previous research (Sadiq et al., 2020; Khalil et al., 2021). This method focused on the potency of the crude and subsequent fractions to reduce the ABTS anion, resulting in a decline in the absorbance when taken on a UV spectrophotometer at 734 nm. ABTS (245 × 10^−3^ M) and K_2_S_2_O_4_ (7 × 10^−3^ M) were primed and placed in the dark for 12 h. Next, this mixture was combined with 0.01 M phosphate to get 0.70 absorbance on the spectrophotometer when measured at 734 nm. All the plant samples were serially diluted, and 300 µL of each was combined with 3.0 mL of standard solution. The percent inhibition results were evaluated in triplicate, and the IC_50_ of all samples was determined.

#### 2.6.3 Hydrogen peroxide assay

In this assay, the crude and subsequent fractions were tested for hydrogen peroxide (H_2_O_2_) antioxidant activity following the published protocol (Yaqoob et al., 2019). All the tested samples were diluted to concentrations ranging from 250 μg/mL to 15.625 μg/mL. In brief, a 50 mM solution of phosphate buffer (pH: 7.4) was prepared accordingly. The 2 mM hydrogen peroxide solution was then made up using the 50 mM phosphate buffer. Aliquots of 100 µL of the extracts or the standard drug (ascorbic acid) were mixed separately with 300 µL of 50 mM phosphate buffer having pH 7.4 in test tubes, and 600 μL of the H_2_O_2_ solution was added to each test tube, mixed, and incubated at 25°C for 10 min. After the incubation period, the absorbance was taken at 230 nm using a spectrophotometer.

### 2.7 *In vivo* assays

#### 2.7.1 Experimental animals

The male Swiss albino mice used in the pharmacological studies weighed 25–30 gm and were 6–8 weeks old when they were acquired from the research laboratory at the National Institutes of Health (NIH) Islamabad, Pakistan. Animals were housed in an appropriate environmental condition and provided with a balanced diet, fresh water, ventilation, and a constant dark/light cycle. The use of the animals in experiments was approved in writing by the Department of Pharmacy at Bacha Khan University, Pakistan, via the Departmental Ethical Committee with ethical approval number DECN/-2022-04 and was conducted according to the Animals By-Laws of 2008 (Scientific Procedure, Issue-I) (Huneif et al., 2023).

#### 2.7.2 Acute toxicity study

Albino mice were used in this study to test the acute toxicity of our plant sample. The animals were randomly assigned to either a control group or an experimental group. Five albino mice were used in each group. Oral administrations of 10–5,000 mg/kg body weight (b.wt) of different samples of *H. aitchisonii* were given to the animals for observation of any toxic effects. Animals were monitored for up to 72 h after dosing for signs of aberrant behavior or mild allergy responses (Zafar et al., 2021).

#### 2.7.3 Analgesic assays

##### 2.7.3.1 Acetic acid-induced writhing test

The analgesic effect of *H. aitchisonii* was tested using the acetic acid-induced writhing test. The plant sample was orally administered at 10 mg/kg, 25 mg/kg, and 50 mg/kg b.wt. Mice were injected with 10 mL/kg of acetic acid (0.6% solution) intraperitoneally (*i.p.*) every 30 min. Group-I received 0.5% of 3 mL/kg Tween-80 as a placebo, whereas Group-II received an injection of 10 mg/kg diclofenac sodium (positive control). The third, fourth, and fifth groups received 10 mg/kg, 25 mg/kg, and 50 mg/kg of *H. aitchisonii* potent fraction, respectively. After being injected with acetic acid, mice were observed to display a variety of writhing behaviors for 15 min. These behaviors included abdominal contractions, limb extension and elongation, and trunk twisting (Muhammad et al., 2023).

##### 2.7.3.2 Formalin test

Average-weight albino mice (25–30 g) were kept in a sterile atmosphere at 23°C ± 2°C with a 12-hour light/dark cycle. All animals had access to free food and drink during the duration of the test. Oral dosages of 10 mg/kg, 25 mg/kg, and 50 mg/kg b.wt of the potent fraction of *H. aitchisonii* were administered. A 20 μL dose of formalin (2.5% v/v in distilled water) was given subcutaneously. Group-I received a negative control injection of 0.5% of 3 mL/kg Tween-80, whereas Group-II received 5 mg/kg morphine as a standard drug. The other groups received tested samples at the doses of 10 mg/kg, 25 mg/kg, and 50 mg/kg. Nociceptive behavior was assumed to explain the formalin-induced licking of paws. Time spent in nociception-related behaviors like biting and licking was tracked. The clock only measured to 30 min. The first 5 min was classified as the neurogenic phase of the nociceptive response, whereas the second 15–30 min was classified as the inflammatory phase (Javed et al., 2022).

##### 2.7.3.3 Hot plate test

The hot plate experimental test is a standard technique for measuring the level of analgesia. The response latencies were measured in this test using the established reported protocol. Albino mice were kept in a glass beaker on a hot plate. Response latency, that is, the time between placing an object and the animal licking, quantified the speed at which the animals reacted to the temperature change. Thirty (30) minutes prior to starting the test, the animals were injected with a tested potent fraction (10 mg/kg, 25 mg/kg, and 50 mg/kg, i.p.) of *H. aitchisonii* or morphine (5 mg/kg, *i.p.*). Mice were monitored before and after 30 min, 60 min, and 90 min following sample injections (Muhammad et al., 2023).

#### 2.7.4 Anti-inflammatory assays

##### 2.7.4.1 Carrageenan-induced inflammation

Paw edema caused by carrageenan was used to measure the anti-inflammatory effect of the tested samples [32]. The animals were divided into five groups. The animals were given only access to water *ad libitum* prior to the experiment. After 30 min, 1% carrageenan (0.05 mL) was administered to the subplantar region of the paw together with diclofenac sodium 50 mg/kg as the standard drug, normal saline as a negative control group, or different test samples. The edema that formed in the paw after the administration of carrageenan was measured using a digital plethysmometer for 1–5 h (Alqahtani et al., 2022).

##### 2.7.4.2 Possible anti-inflammatory mechanism

We used various phlogistic agents like histamine, prostaglandin E_2_ (PGE_2_), bradykinin, and leukotriene to evaluate the possible mechanism. Briefly, in this study, we gave an intraperitoneal injection of 10% dimethylsulfoxide (DMSO), montelukast (lipoxygenase inhibitor), 100 mg/kg, HOE 140 (bradykinin inhibitor), chlorpheniramine maleate (antihistaminic), 1 mg/kg, celecoxib (cyclooxygenase inhibitor), 50 mg/kg, or the tested potent fraction (50 mg/kg) to male BALB/c mice (25–30 g). Subplantar injections of 10 mg/mL leukotriene, 1 mg/mL histamine, 20 mg/mL bradykinin, or 0.1 mg/mL PGE_2_ were used to produce paw edema after 1 h. The paw volume of each mouse was determined at the first to the fifth hour after the injection of various irritants (inflammatory agents) into the subplantar region (Javed et al., 2022).

### 2.8 Antioxidant *ex vivo* analysis

#### 2.8.1 Estimation of GSH reductase, SOD, MDA, and catalase

For the GSH reductase *ex vivo* activity reaction, a mixture of phosphate buffer, oxidized GSH, EDTA, and NADPH was prepared with distilled water. After preparation of the reaction mixture, a tissue homogenate was mixed for optical density observation at an absorbance of 340 nm for 2 min at 30 s intervals. The resulting enzymatic activity was expressed in moles of the NADPH oxidized/min/mg protein (Davidson and Hird, 1964). Similarly, in the superoxide dismutase assay, a reaction mixture containing homogenate with carbonate buffer and epinephrine was prepared, and each sample absorbance was recorded at 480 nm on a UV spectrophotometer at an interval of 15 s for 2 min (Misra and Fridovich, 1976). For thiobarbituric acid reactive substances (TBARS)–malonaldehyde level lipid peroxidation indirect measurement, Malondialdehyde (MDA) was used and can be determined by reaction with thiobarbituric acid. Homogenate was placed in test tubes, and TBA, trichloroacetic acid, and 0.25 M hydrochloric acid were added to homogenate. The solution was shaken and allowed to sit for 15 min without any disturbance, and then the mixture was placed in an ice bath for cooling. The solution was centrifuged for 10 min after cooling. Then, the upper layer of solution was collected and assessed at 532 nm on the spectrophotometer (Ohkawa et al., 1979). The catalase (CAT) levels were measured following the procedure of Aebi (1984). Hydrogen peroxide was added to the test sample solution, which consisted of tissue homogenate and phosphate buffer, and absorbance changes at 240 nm were followed for 30 s at 15 s intervals.

### 2.9 Computational studies

Computational studies are the fundamental tools to detect the binding potential of identified compounds against targeted protein moieties. They involve the calculation of free binding energy between the ligand and the protein, which gives valuable information about the nature and strength of any interaction. These studies also explore the conformational changes that occur in the targeted protein when approached by ligands. The predicted binding posture of the ligand could be utilized later to identify the allosteric sites for future target exploitation. All the structures of identified compounds were drawn in ChemDraw 20.0 software and saved in the mol.file format. Meanwhile, structures of targeted protein COX-2 (1CX2 co-crystallized with SC-558) were downloaded from the RSCB Protein Data Bank and saved in the pdb file format. The structures of ligands and targeted proteins were modified after the removal of co-crystallized ligands, water molecules, and the addition of polar hydrogen atoms and resaved in the pdb format. Docking studies were carried out through AutoDock Vina software interlinked with PYRX. After docking, the best binding posture was identified as that with the lowest binding energy and highest binding affinity for the protein. The results were amplified and displayed through Discovery Studio Visualizer and PyMOL.

### 2.10 Statistical data analysis and estimation of IC_50_ values

We determined the concentration of each sample that resulted in a 50% inhibition of substrate hydrolysis (IC_50_) using Microsoft Excel. The same approach was used to determine the IC_50_ in free radical assays, including DPPH, ABTS, and H_2_O_2_ (Alshehri et al., 2024). Data values were represented as mean ± SEM. for all tests, which were carried out in triplicate. GraphPad Prism Software, USA, was used to perform an ANOVA followed by a Bonferroni test to compare the test group with the positive control group. Statistics were deemed significant for *p-*values under 0.05. All *in vitro* test results are shown as mean ± SEM, with *n* = 3. The *p*-values are compared with the reference drug, such as * = *p* < 0.05, ** = *p* < 0.01, and *** = *p* < 0.001.

## 3 Results

### 3.1 Phytochemistry

**FIGURE 1:**
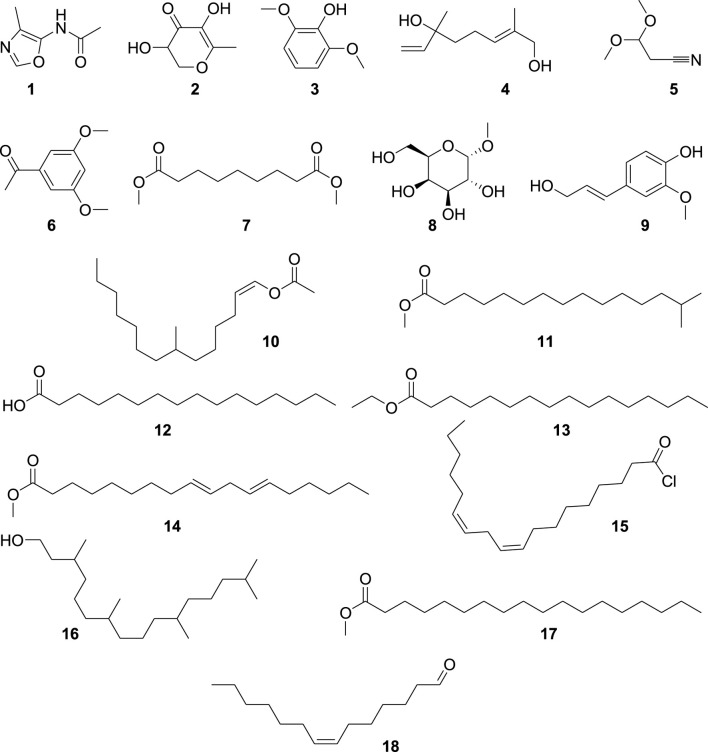
Structures of the identified compounds in *H. aitchisonii.*

**FIGURE 2:**
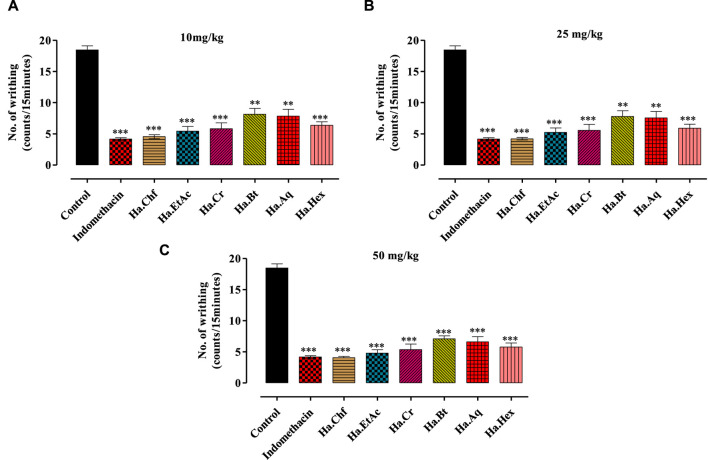
Effects of different concentrations of acetic acid on *H. aitchisonii* test findings. A difference in writhing responses (means ± SEM, *n* = 5) was observed between the control (DMSO), different fractions of *H. aitchisonii*, and the acetyl salicylic acid (ASA) positive control group. The values were very different between the tested and control groups (**p < 0.001, ***p < 0.0001). Ha, *H. aitchisonii*; Cr, crude; Hex, hexane; Chf, chloroform; EtAc, ethyl acetate; Bt, butanol; Aq, aqueous fraction. **(A)**: 10 mg/kg; **(B)**: 25 mg/kg; **(C)**: 50 mg/kg.

We analyzed Ha.Cr using GC-MS (Figure 1) and found 18 different compounds, which are displayed in Figure 2. GC-MS detection depends on peak-to-library matches based on spectral peak, mass, and fragmentation pattern. Consequently, it is possible for two molecules to have the same mass spectrum and fragmentation pattern, although this is very rare. Additionally, new chemicals inside a plant will remain unidentified if their information is not placed in a GC-MS library. Supplementary Table S1 provides the GC-MS analysis results. Compound 18 (Supplementary Figure S1), with a retention time of 44.379 min, is one of the most prominent compounds in the GC-MS chromatogram (Figure 1).

### 3.2 *In vitro* anti-inflammatory assay results

#### 3.2.1 COX-2 results

The % inhibitions of COX enzymes in different fractions of *H. aitchisonii* are summarized in Table 1. There was a dose–response relationship between the samples and COX-2 inhibition. The observed % inhibition was determined on concentrations ranging from a maximum of 250 μg/mL to a minimum of 15.625 μg/mL across all the samples. The standard drug celecoxib had similar results, with 95.24% inhibition at 250 μg/mL and 81.88% at 15.625 μg/mL. Ha.Chf (88.18% inhibition at 250 μg/mL) and Ha.EtAc (80.26% inhibition at 250 μg/mL) showed the closest activity to that of the reference drug in our plant samples. Ha.Chf and Ha.EtAc provided very effective, with IC_50_ values of 0.804 μg/mL and 2.62 μg/mL, respectively. The median effective dose (IC_50_) for a reference drug was 0.594 μg/ml, as shown in Table 1.

**TABLE 1:** *In vitro* assays of the crude and subsequent fraction of *H. aitchisonii* against COX-2 and 5-LOX enzymes.

Sample name	Conc. (µg/mL)	COX-2		5-LOX	
		% Inhibition	IC_50_ (µg/mL)	% Inhibition	IC_50_ (µg/mL)
Ha.Cr	250	89.10 ± 1.82*	1.62	88.63 ± 1.50*	3.46
	125	86.19 ± 1.56**		84.17 ± 0.15**	
	62.5	82.34 ± 1.64**		78.10 ± 0.14***	
	31.25	76.78 ± 0.62***		72.89 ± 0.17***	
	15.625	71.10 ± 0.80***		66.78 ± 0.72***	
Ha.Hex	250	81.61 ± 1.32***	4.78	83.45 ± 1.22**	4.59
	125	77.78 ± 0.90***		78.88 ± 0.22***	
	62.5	73.45 ± 0.43***		73.77 ± 0.11***	
	31.25	65.45 ± 0.43***		68.89 ± 0.13***	
	15.625	61.23 ± 0.13***		61.19 ± 0.13***	
Ha.Chf	250	88.18 ± 1.74**	0.804	82.15 ± 0.14***	1.70
	125	85.17 ± 1.56***		80.14 ± 0.86***	
	62.5	83.23 ± 1.32***		76.17 ± 0.68***	
	31.25	77.12 ± 0.90***		71.90 ± 0.96***	
	15.625	73.12 ± 0.90***		65.45 ± 0.43***	
Ha.EtAc	250	80.26 ± 1.96***	2.62	84.19 ± 1.62**	3.52
	125	79.56 ± 1.76***		79.10 ± 1.10***	
	62.5	74.32 ± 1.52***		73.44 ± 0.42***	
	31.25	68.89 ± 0.13***		68.89 ± 0.19***	
	15.625	63.13 ± 0.49***		64.14 ± 0.10***	
Ha.Bt	250	75.32 ± 2.87***	19.37	79.37 ± 1.04***	15.22
	125	67.12 ± 0.54***		72.37 ± 0.54***	
	62.5	62.79 ± 1.08***		65.30 ± 2.61***	
	31.25	55.79 ± 1.88***		58.42 ± 1.05***	
	15.625	47.20 ± 0.47***		50.52 ± 2.52***	
Ha.Aq	250	88.88 ± 0.89**	8.24	78.34 ± 1.16***	8.91
	125	83.54 ± 3.60***		72.88 ± 0.92***	
	62.5	75.01 ± 1.97***		66.67 ± 0.23***	
	31.25	67.68 ± 0.22***		61.11 ± 0.19***	
	15.625	59.82 ± 1.95***		55.78 ± 0.92***	
Celecoxib	250	95.24 ± 0.90	0.594	---	---
	125	92.45 ± 0.43			
	62.5	88.88 ± 0.82			
	31.25	85.12 ± 0.14			
	15.625	81.88 ± 0.44			
Montelukast	250	---	---	93.12 ± 0.56	0.762
	125			89.45 ± 0.43	
	62.5			84.44 ± 0.40	
	31.25			81.90 ± 0.92	
	15.625			78.12 ± 0.20	

The effect of the tested various fractions on COX-2 and 5-LOX inhibition. The values were expressed as mean ± SEM. *p < 0.05, **p < 0.01, and ***p < 0.001. Data were analyzed via two-way ANOVA followed by Bonferroni post-test compared to standard drugs (celecoxib for COX-2 and montelukast for 5-LOX). Ha, *H. aitchisonii*; Cr, crude; Hex, hexane; Chf, chloroform; EtAc, ethyl acetate; Bt, butanol; Aq, aqueous fraction.

### 3.3 5-LOX results

Table 1 compiles the IC_50_ values and % inhibitions of all tested fractions of the 5-LOX assay. The pattern of responses seen was consistent with the results of a COX-2 test, with a few notable outliers. The % inhibition of Ha.Chf at the maximum dose of 250 μg/mL was comparable to the standard drug montelukast. Ha.Chf exhibited 82.15% inhibition, and montelukast exhibited 93.12% inhibition. Similarly, Ha.EtAc inhibited 5-LOX by 78.34% at a concentration of 250 μg/mL. The Ha.Chf and Ha.EtAc were the two most powerful plant-based extracts. We measured IC_50_ values of 3.52 μg/mL and 15.22 μg/mL for Ha.Chf and Ha.EtAc, respectively. The IC_50_ value of the reference medication was determined to be 0.762 μg/mL after being compared to our samples.

### 3.4 Antioxidant assay results

#### 3.4.1 ABTS scavenging assay

In ABTS free radical scavenging activity, the Ha.Chf was the most active and potent fraction and exhibited 93.08% ± 1.04, 86.45% ± 0.90, 80.58% ± 0.63, 75.40% ± 0.20, and 70.80% ± 0.90 inhibition with an IC_50_ value of 3.25 μg/mL. The second highest activity was displayed by the Ha.EtAc with a percent inhibition of 6.91%–66.76% from concentrations of 250 μg/mL, 125 μg/mL, 62.5 μg/mL, 31.25 μg/mL, and 15.625 μg/mL and an IC_50_ of 3.32 μg/mL. The remaining fractions also showed good to moderate activity against ABTS free radicals. The standard drug ascorbic acid displayed 91.51% inhibition at the highest concentration (250 μg/mL) and 72.72% at the lowest concentration (15.625 μg/mL) with an IC_50_ value of 1.82, as shown in Table 2.

**TABLE 2:** *In vitro* assays of crude and subsequent fractions of *H. aitchisonii* against ABTS, DPPH, and H_2_O_2_.

Sample name	Conc. (µg/mL)	% Scavenging ABTS	IC_50_ (µg/mL)	% Scavenging H_2_O_2_	IC_50_ (µg/mL)	% Scavenging DPPH	IC_50_ (µg/mL)
Ha.Cr	250	84.23 ± 0.22***	5.48	76.29 ± 0.43***	11.52	82.36 ± 0.57***	3.91
	125	80.45 ± 0.90***		69.56 ± 0.45***		77.85 ± 2.24***	
	62.5	74.90 ± 0.60***		63.54 ± 0.46***		72.08 ± 0.47***	
	31.25	66.00 ± 0.30***		58.57 ± 0.84***		67.90 ± 0.96***	
	15.625	61.90 ± 0.45***		53.56 ± 1.73***		62.28 ± 0.57***	
Ha.Hex	250	82.88 ± 0.89***	8.21	73.84 ± 0.10***	15.40	83.36 ± 0.49***	5.23
	125	78.54 ± 0.60***		67.15 ± 0.14***		81.34 ± 0.55***	
	62.5	73.01 ± 0.97***		61.56 ± 0.74***		76.39 ± 0.49***	
	31.25	67.68 ± 0.22***		55.12 ± 0.34***		71.47 ± 0.52***	
	15.625	59.82 ± 0.95***		51.31 ± 2.15***		63.44 ± 0.55***	
Ha.Chf	250	93.08 ± 1.04^ns^	3.25	86.63 ± 0.64^ns^	4.57	89.37 ± 0.54**	3.63
	125	86.45 ± 0.90^ns^		80.45 ± 0.55^ns^		84.44 ± 0.50*	
	62.5	80.58 ± 0.63^ns^		74.53 ± 0.41^ns^		77.51 ± 0.72***	
	31.25	75.40 ± 0.20^ns^		69.42 ± 0.46^ns^		72.28 ± 0.61***	
	15.625	70.80 ± 0.90^ns^		63.68 ± 0.64^ns^		67.46 ± 0.62***	
Ha.EtAc	250	86.91 ± 1.30***	3.32	81.85 ± 0.18**	5.59	88.53 ± 0.20**	3.52
	125	81.26 ± 1.27***		76.59 ± 0.30***		83.62 ± 0.17***	
	62.5	76.00 ± 0.30***		71.75 ± 0.14**		77.42 ± 0.11***	
	31.25	69.54 ± 0.50***		66.47 ± 0.49***		71.20 ± 0.15***	
	15.625	66.76 ± 0.58***		61.12 ± 0.34***		67.35 ± 0.18***	
Ha.Bt	250	74.4 ± 0.68***	19.65	69.65 ± 1.32***	25.66	76.7 ± 0.66***	15.38
	125	66.2 ± 0.73***		64.42 ± 0.43***		71.3 ± 1.11***	
	62.5	61.0 ± 0.33^***^		58.25 ± 1.40***		65.5 ± 1.04***	
	31.25	56.4 ± 0.63^***^		52.22 ± 1.28***		57.2 ± 0.57***	
	15.625	46.9 ± 0.42***		45.03 ± 0.48***		49.9 ± 0.65***	
Ha.Aq	250	77.42 ± 0.68***	12.76	84.44 ± 0.55^ns^	6.76	79.77 ± 0.66^***^	8.52
	125	67.21 ± 0.73***		81.39 ± 0.49^ns^		71.30 ± 1.11***	
	62.5	62.00 ± 0.33***		73.56 ± 0.45^ns^		67.52 ± 1.04***	
	31.25	57.42 ± 0.63***		68.52 ± 0.66*		61.21 ± 0.57***	
	15.625	53.56 ± 1.06***		58.30 ± 0.64^ns^		56.56 ± 0.74***	
Ascorbic acid	250	91.51 ± 0.62	1.82	87.10 ± 0.20	4.32	94.58 ± 0.69	2.17
	125	86.65 ± 0.70		83.40 ± 1.12		88.68 ± 0.42	
	62.5	81.25 ± 0.55		76.90 ± 0.88		84.46 ± 0.72	
	31.25	77.37 ± 0.69		73.88 ± 0.44		79.50 ± 0.71	
	15.625	72.72 ± 0.51		61.90 ± 1.10		74.47 ± 0.59	

The effect of tested various fractions on ABTS, DPPH, and H_2_O_2_% inhibition. The values are expressed as mean ± SEM. *p < 0.05, **p < 0.01, and ***p < 0.001 compared to standard drugs (ascorbic acid). Data were analyzed via two-way ANOVA followed by the Bonferroni post-test. Ha, *H. aitchisonii*; Cr, crude; Hex, hexane; Chf, chloroform; EtAc, ethyl acetate; Bt, butanol; Aq, aqueous fraction.

#### 3.4.2 DPPH scavenging results

In the DPPH free radical scavenging assay, the Ha.Chf fraction exhibited 89.37% ± 0.54, 89.37% ± 0.54, 84.44% ± 0.50, 77.51% ± 0.72, 72.28% ± 0.61, and 67.46% ± 0.62 inhibition at concentrations of 15.625–250 μg/mL with an IC_50_ of 3.63 μg/mL. The Ha.EtAc exhibited 88.53% ± 0.20, 83.62% ± 0.17, 77.42% ± 0.11, 71.20% ± 0.15, and 67.35% ± 0.18% inhibition at concentrations of 250 μg/mL, 125 μg/mL, 62.50 μg/mL, 31.25 μg/mL, and 15.625 μg/mL with an IC_50_ value of 3.52 μg/mL, respectively. The ascorbic acid was used as standard with an IC_50_ value of 2.17 μg/mL. All the other fractions, like Ha.Cr, Ha.Hex, Ha.Bt, and Ha.Aq, exhibited 82.36% ± 0.57, 83.36% ± 0.49, 76.7% ± 0.66% and 79.77% ± 0.66% inhibition at the highest concentration, which is 250 μg/mL, with IC_50_ values of 3.91 μg/mL, 5.23 μg/mL, 15.38 μg/mL, and 8.52 μg/mL, respectively (Table 2).

#### 3.4.3 H_2_O_2_ scavenging results

The results of *in vitro* H_2_O_2_ free radical scavenging assay of various fractions of *H. aitchisonii* are displayed in Table 2. All the tested fractions display dose-dependent H_2_O_2_ free radical scavenging results. The concentration was the same as in previous ABTS and DPPH scavenging assays. The same procedure was followed for the standard drug, ascorbic acid, which showed 87.10% inhibition at 250 μg/mL and 61.90% at 15.625 μg/mL. In our tested samples, the highest results were shown again by Ha.Chf (86.63% inhibition at the highest dose) followed by Ha.EtAc (81.85% inhibition). The IC_50_ values exhibited by Ha.EtAc and Ha.Chf are 5.59 μg/mL and 4.57 μg/mL, respectively. In contrast, the standard drug IC_50_ was 4.32 μg/mL.

### 3.5 *In vivo* assay results

#### 3.5.1 Acute toxicity observations

There were no deaths or abnormal behaviors seen in the experimental animals throughout the period of acute study when they were given doses of up to 3,000 mg/kg b.wt. A dosage of 3,000 mg/kg from *H. aitchisonii* samples is regarded as safe based on acute toxicity tests. Dosage information for the animals is shown in Table 3.

**TABLE 3:** Acute toxicity study of *H. aitchisonii*.

Groups	Animals per group	*H. aitchisonii* (conc. µg/mL)
1	5	10
2	5	25
3	5	50
4	5	100
5	5	200
6	5	500
7	5	1,000
8	5	2,000
9	5	3,000
10	5	5,000

#### 3.5.2 Analgesic assay results

##### 3.5.2.1 Acetic acid-induced writhing test

A dose-dependent analgesic effect was observed in an acetic acid-induced writhing test. Doses of 10 mg/kg, 25 mg/kg, and 50 mg/kg b.wt were used to test each fraction. The maximum analgesic effect was shown by the Ha.Chf and Ha.EtAc fractions at a dosage of 25 mg/kg b.wt, exceeding that of the standard drug indomethacin (Figure 2C). At 10 mg/kg, the standard drug displayed a mean writhes inhibition of 77.42%. Ha.Chf was more effective than the standard drug with 77.96% inhibition of mean writhes. The ethyl acetate fraction (Ha.EtAc) at the highest dose demonstrated the greatest analgesic effect (74.01%). Figures 2A, B demonstrate that all the other fractions exhibit good to moderate peripheral analgesic effects at 25 mg/kg b.wt and 10 mg/kg b.wt.

##### 3.5.2.2 Formalin test

In the formalin test, mice that were injected with 2% formalin intra-plantar (i.p.) exhibited a normal two-phase licking response. In the negative control group, in early phase the paw licking was 56.21 ± 0.42 s (between 0 min and 5 min), while in the late phase the paw licking was 78.02 ± 0.45 (between 15 min and 30 min). The effects of pretreatment with 10 mg/kg *i.p.*, 25 mg/kg *i.p.*, and 50 mg/kg *i.p.* of various plant samples were tested. Excellent results were seen with Ha.Chf, and suppression of licking activity in both phases was evident at 50 mg/kg (Table 4). Inhibitions in the early and late stages of paw licking were 86.47% and 81.49%, respectively. Both neurogenic pain (early phase, 86.80% inhibition) and inflammatory pain (late phase, 93.81% inhibition) responded well to a 5 mg/kg i.p. injection of morphine. Thus, our Ha.Chf sample was comparably active to the standard drug in the first stages. Ha.EtAc showed similar results, with 65.10%, 75.95%, and 83.81% inhibition at 10 mg/kg, 25 mg/kg, and 50 mg/kg in the early phase and 62.25%, 69.78%, and 75.81% inhibition in the late phase. Percent inhibition was measured via the following formula:

$$\%\text{Inhibition}=\left\{\left(\text{Wcg}-\text{Wtg}\right)\times 100\right\}/\text{Wcg}.$$

**TABLE 4:** Effect of various fractions of *H. aitchisonii* on formalin-induced pain in mice.

Sample	Dose mg/kg b.wt	Total time spent in licking			
		0–5 min	% Inhibition	15–30 min	% Inhibition
Negative control	-	56.21 ± 0.42	-	78.02 ± 0.45	-
Hd.Cr	10	27.32 ± 0.40	51.39***	40.81 ± 0.20	47.69***
	25	19.74 ± 0.76	64.88***	29.29 ± 0.47	62.45***
	50	12.56 ± 0.44	77.66***	19.90 ± 0.96	74.49***
Hd.Hex	10	47.31 ± 0.31	15.83^ns^	68.84 ± 0.30	11.77^ns^
	25	37.62 ± 0.82	33.07**	58.08 ± 0.47	25.56*
	50	29.77 ± 0.53	47.04***	45.42 ± 0.46	41.78**
Hd.Chf	10	15.02 ± 0.20	73.28***	24.76 ± 0.71	68.26***
	25	10.32 ± 0.30	81.64***	17.90 ± 0.96	77.06***
	50	7.60 ± 0.92	86.47***	14.44 ± 0.58	81.49***
Hd.EtAc	10	19.62 ± 0.60	65.10***	29.45 ± 0.90	62.25***
	25	13.52 ± 0.42	75.95***	23.58 ± 0.63	69.78***
	50	9.10 ± 0.12	83.81***	18.87 ± 0.85	75.81***
Hd.Bt	10	41.72 ± 0.52	25.77^ns^	61.76 ± 0.61	20.84^ns^
	25	35.50 ± 0.60	36.84**	52.49 ± 0.60	32.72*
	50	27.32 ± 0.64	51.40***	42.45 ± 0.90	45.59***
Hd.Aq	10	33.40 ± 0.20	40.58*	51.58 ± 0.63	33.89^ns^
	25	26.50 ± 0.52	52.85**	42.10 ± 0.60	46.04**
	50	20.40 ± 0.52	63.71***	32.51 ± 0.54	58.33***
Morphine	5 mg/kg	7.42 ± 0.72	86.80***	4.83 ± 0.60	93.81***

The values are expressed as mean ± SEM. *p < 0.05, **p < 0.01, and ***p < 0.001. Data were analyzed via two-way ANOVA followed by the Bonferroni post-test compared to standard drugs (morphine). ns, not significant; Ha, *H. aitchisonii*; Cr, crude; Hex, hexane; Chf, chloroform; EtAc, ethyl acetate; Bt, butanol; Aq, aqueous fraction.

##### 3.5.2.3 Hot plate test

Table 5 summarizes the pain-relieving effects seen in the hot plate test. Compared to the positive control group (morphine), the latency time for the Ha.Chf was shown to increase with increased dose. The mean response times at 15 min after the administration of Ha.Chf were 8.53 ± 0.62, 10.90 ± 0.10, and 12.52 ± 0.54 s for dosages of 10, 25, and 50 mg/kg, respectively. Mean response times at the final time point of 60 min were 8.10 ± 0.20, 9.50 ± 0.52, and 11.80 ± 0.22 s for the 10, 25, and 50 mg/kg b.wt dosages, respectively. The standard drug morphine at a dosage of 5 mg/kg was reported to have an initial reaction time at 15 min of 12.22 ± 0.20 s and at 60 min of 12.10 ± 0.32 s. Similar results were found for Ha.EtAc, with a mean response time of 8.20 ± 0.26, 9.86 ± 0.50, and 11.98 ± 0.32 s at 10, 25, and 50 mg/kg, respectively, after an initial dosage of 15 min. Ha.EtAc response times at 60 min were measured to be 7.88 ± 0.44, 8.80 ± 0.44, and 11.20 ± 0.22 s for the same concentrations. Table 5 also displays excellent to moderate results for the other fractions, which include Ha.Cr, Ha.Hex, Ha.Bt, and Ha.Aq.

**TABLE 5:** Analgesic activities of crude and various fractions of *H. aitchisonii* following the hot plate model.

Sample	Dose mg/kg b.wt	Reaction time on the hot plate in seconds			
		15	30	45	60
Negative control	-	3.91 ± 0.52	4.94 ± 0.22	3.32 ± 0.52	2.72 ± 0.40
Ha.Cr	10	6.45 ± 0.87^ns^	5.52 ± 0.62^ns^	4.67 ± 0.47^ns^	3.30 ± 0.74^ns^
	25	7.52 ± 0.50*	6.41 ± 0.87^ns^	5.50 ± 0.62^ns^	4.69 ± 0.49^ns^
	50	8.32 ± 0.44***	8.63 ± 0.39**	7.24 ± 0.58*	6.15 ± 0.73^ns^
Ha.Hex	10	3.48 ± 0.57^ns^	3.34 ± 0.92^ns^	2.65 ± 0.32^ns^	2.24 ± 0.55^ns^
	25	4.15 ± 0.62^ns^	3.15 ± 0.74^ns^	3.48 ± 0.57^ns^	3.14 ± 0.92^ns^
	50	6.45 ± 0.64^ns^	5.86 ± 0.36^ns^	5.62 ± 0.78^ns^	4.28 ± 0.45^ns^
Ha.Chf	10	8.53 ± 0.62***	8.10 ± 0.20*	7.90 ± 0.30*	6.52 ± 0.52^ns^
	25	10.90 ± 0.10***	9.50 ± 0.52***	9.10 ± 0.32***	8.60 ± 0.28**
	50	12.52 ± 0.64***	11.80 ± 0.22***	10.60 ± 0.20***	10.10 ± 0.10***
Ha.EtAc	10	8.20 ± 0.26***	7.88 ± 0.44*	7.10 ± 0.22*	5.96 ± 0.76^ns^
	25	9.86 ± 0.50***	8.80 ± 0.44**	8.20 ± 0.46**	8.22 ± 0.28*
	50	11.98 ± 0.32***	11.20 ± 0.22***	10.10 ± 0.52**	9.62 ± 0.52**
Ha.Bt	10	3.66 ± 0.64^ns^	4.50 ± 0.92^ns^	3.50 ± 0.52^ns^	3.73 ± 0.71^ns^
	25	5.80 ± 0.32^ns^	4.78 ± 0.60^ns^	4.50 ± 0.60^ns^	4.52 ± 0.82^ns^
	50	6.96 ± 0.90**	5.60 ± 0.20^ns^	5.90 ± 0.60^ns^	5.26 ± 0.53^ns^
Ha.Aq	10	4.66 ± 0.81^ns^	4.548 ± 0.43^ns^	4.50 ± 0.30^ns^	3.55 ± 0.96^ns^
	25	5.55 ± 0.81^ns^	5.69 ± 0.80^ns^	4.81 ± 0.44^ns^	4.13 ± 0.27^ns^
	50	6.92 ± 0.82**	5.80 ± 0.60^ns^	5.59 ± 0.33^ns^	5.68 ± 0.34^ns^
Morphine	5	12.22 ± 0.20***	12.10 ± 0.32***	11.44 ± 0.42***	11.16 ± 0.10***

The data are shown as mean ± SEM (*n* = 5) for simplicity. The asterisks represent statistically significant differences from the baseline condition: Student’s *t*-test was used to analyze the data. ^∗^ *p* < 0.05; ^∗∗^ *p* < 0.01, ∗∗∗*p* < 0.001. ns, not statistically significant; Ha, *H. aitchisonii*; Cr, crude; Hex, hexane; Chf, chloroform; EtAc, ethyl acetate; Bt, butanol; Aq, aqueous fraction.

#### 3.5.3 Anti-inflammatory assay results

##### 3.5.3.1 Carrageenan-induced paw edema

**FIGURE 3:**
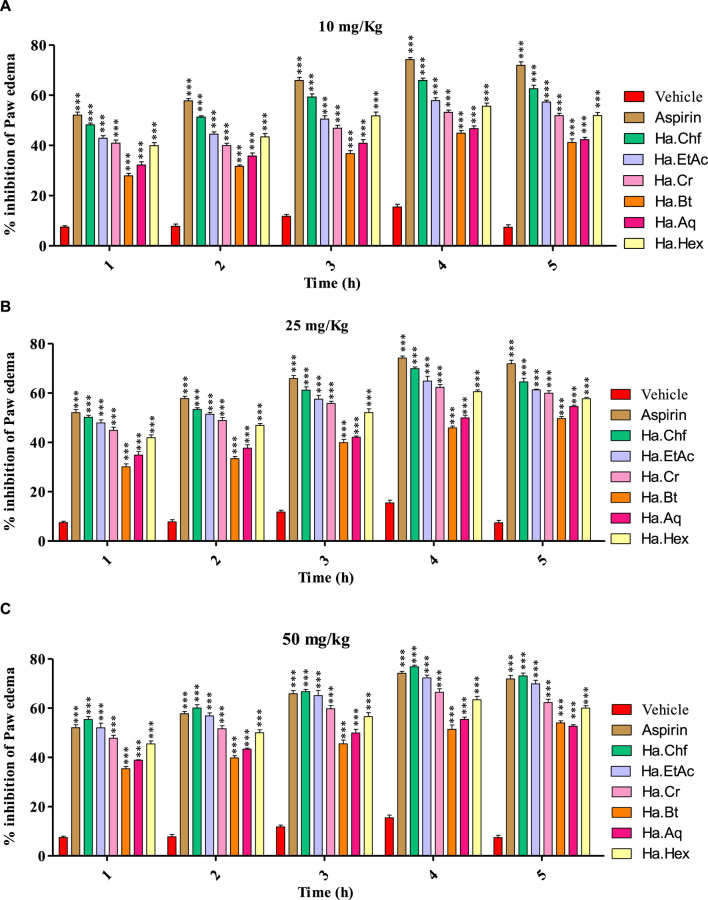
Carrageenan-induced paw edema test of the *H. aitchisonii* and subsequent fractions. The two-way ANOVA was followed by the Bonferroni test. Values were significantly (∗∗∗*p* < 0.001) different from the control group. Ha, *H. aitchisonii*; Cr, crude; Hex, hexane; Chf, chloroform; EtAc, ethyl acetate; Bt, butanol; Aq, aqueous fraction.

All samples (10 mg/kg, 25 mg/kg, and 50 mg/kg b.wt) showed good to moderate activity in carrageenan-induced inflammation, as shown in (Figures 3A–C). Figure 3 shows that the Ha.Chf and Ha.EtAc fractions both have great anti-inflammatory potential. At the maximum dosage (50 mg/kg), Ha.Chf showed an anti-inflammatory potential of 55.40% after the first hour and remained active until the fifth hour of observations (78.80%), which was more active than the standard drug aspirin (52.10%–74.20%, from the first to the fourth hour). The Ha.EtAc fraction demonstrated activity at 50 mg/kg b.wt (52.02%–74.02%, first to the fourth hour). The other *H. aitchisonii* tested samples ranged from good to moderate inhibition. Carrageenan-induced inhibitions of different *H. aitchisonii* fractions were ranked as follows: Ha.Chf > Ha.EtAc > Ha.Cr > Ha.Hex > Ha.Aq > Ha.Bt.

##### 3.5.3.2 Involvement of the anti-inflammatory mechanism

###### 3.5.3.2.1 Effect of the potent fraction on paw edema induced by various phlogistic agents

**FIGURE 4:**
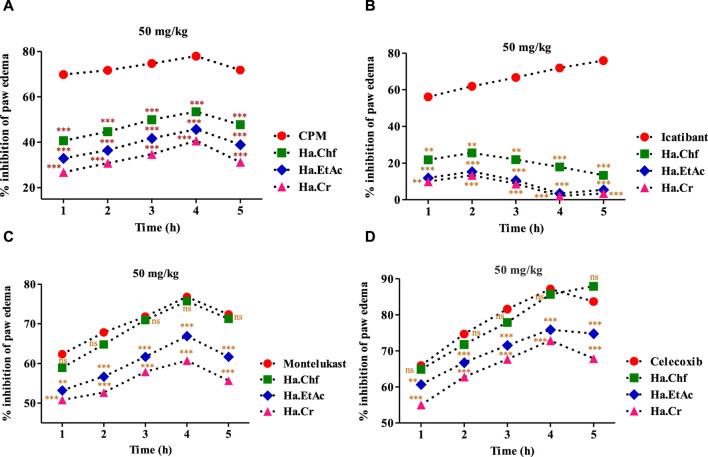
Various phlogistic agents that use an anti-inflammatory mechanism. **(A)** In a mouse model of paw edema triggered by histamine, the % inhibition caused by tested powerful fractions (50 mg/kg); **(B)** % inhibition caused by the most potent fractions (50 mg/kg) in a mouse model of bradykinin-induced paw edema; **(C)** the same for a model of PGE_2_-induced paw edema; and **(D)** the same for a model of leukotriene-induced paw edema. The % points indicate a mean value for a sample size of eight mice. Two-way ANOVA post-test analysis was performed on the data. n.s., not significant, *p > 0.05, ** *p* = 0.01, *** *p =* 0.001. Ha, *H. aitchisonii*; Cr, crude; Chf, chloroform; EtAc, ethyl acetate fraction.

In the histamine-induced assay, at 1 mg/kg b.wt, chlorpheniramine maleate substantially reduced histamine-induced inflammation during the first hour (69.77%) and maintained this effect through the fourth hour. Similar results were seen with the Ha.Chf tested fraction, which showed considerable inhibitory potential (40.66%) in the first hour after administering a dose of 50 mg/kg. This effect persisted for another 3 h. A similar reduction of inflammation brought on by histamine administration (32.90%) was seen with the Ha.EtAc tested fraction at a dose of 50 mg/kg (Figure 4A). Similarly, in the first hour, Ha.Cr showed an inhibition of 26.78%, and it showed an inhibition of 40.66% in the fourth hour. Furthermore, following injection of bradykinin (20 mg/mL), the average volume of paw edema in mice pretreated with 50 mg/kg b.wt was determined at the first, second, third, fourth, and fifth hours. In inflammation generated by bradykinin, the most active fractions studied were less effective than the positive control. At the second hour post-bradykinin injection, Ha.Chf exhibited 25.56% inhibition, whereas Ha.EtAc and Ha.Cr showed only 15.40% and 13.40% inhibition, respectively, which was significantly lower than the positive control HOE 140, as shown in Figure 4B. A similar increase in paw edema was seen after PGE_2_ (0.01 mg/mL) administration. Treatment with Ha.Chf, Ha.EtAc, and Ha.Cr (50 mg/kg), as well as celecoxib (50 mg/kg), considerably reduced the inflammatory responses to PGE_2_. Ha.Chf considerably decreased PGE_2_-induced paw edema, starting at a 64.88% reduction after the first hour and reaching a peak at the fifth hour with an 87.90% reduction. Results for Ha.EtAC and Ha.Cr were also encouraging (60.67%–75.89%; 1–4 h; 55.02%–72.89%; 1–4 h), and they held up well even after 5 h. The maximum percentage reduction of paw inflammation was seen with celecoxib (first to the fifth hour; 65.92%–83.67%; Figure 4C). Likewise, at 50 mg/kg b.wt, the most powerful fraction evaluated showed anti-inflammatory efficacy in a leukotriene-induced inflammatory response (Figure 4D). In a dose-dependent manner, the tested samples suppressed the edema induced by leukotriene (10 mg/mL). At the third hour after leukotriene administration, Ha.EtAc and Ha.Cr showed significant anti-inflammatory activity with 66.88% and 60.72%, respectively, significantly closer to that of standard drug. Ha.Chf showed greatest inhibition (58.90%–75.67%; first to the fourth hour). The positive control drug montelukast reduced paw inflammation by 76.78% activity after the fourth hour.

#### 3.5.4 Effect of *H. aitchisonii* on antioxidant levels

##### 3.5.4.1 LPS induced alterations in GSH, SOD, MDA, and CAT levels

**FIGURE 5:**
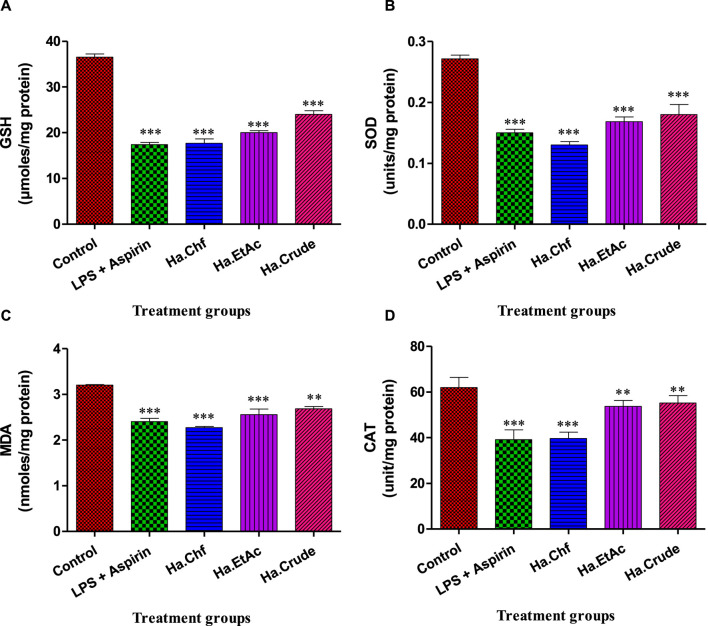
Effect of *H.* aitchisonii on antioxidant levels of GSH **(A)**, SOD **(B)**, MDA **(C)**, and CAT **(D)**. Values are shown as mean ± SEM. (*n* = 6). One-way ANOVA was followed by Dunnett's multiple comparison test, and data are represented as significant values as ***p < 0.001, **p < 0.01. LPS, lipopolysaccharide; Ha, *H. aitchisonii*; Cr*,* crude; Chf, chloroform; EtAc, ethyl acetate fraction.

When compared to the control group, the lipopolysaccharide (LPS)-treated mice had substantially lower levels of GSH (*p* < 0.001). The aspirin-treated mice had considerably higher levels of GSH than the LPS-treated animals (*p* < 0.001). In comparison to LPS-treated mice, animals treated with a 25 mg/kg dose of *H. aitchisonii* showed a statistically significant increase in GSH levels (*p* < 0.001), as shown in Figure 5A. The level of superoxide dismutase (SOD) was also considerably lower in the LPS-treated group than in the control group (*p* < 0.001). The SOD levels of the aspirin-treated mice group were considerably higher than those of the LPS-treated mice group (*p* < 0.001). At the same dosage, *H. aitchisonii* substantially increased SOD activity (*p* < 0.001) (Figure 5B). The level of MDA in LPS-treated animals was also considerably higher than in control mice (*p* < 0.001). The MDA level in the aspirin-treated mice group was considerably lower than in the LPS-treated mice group (*p* < 0.001). While *H. aitchisonii* dosages of 25 mg/kg b.wt considerably (*p* < 0.001) reduced MDA levels in mice compared to LPS-treated animals, the effect was less dramatic than that seen in the aspirin-treated mice (Figure 5C). When compared to a control group of mice, the CAT level in the experimental group was also considerably lower (*p* < 0.001). The CAT enzyme was considerably (*p* < 0.001) increased in the aspirin-treated mice group compared to the LPS-treated mice group. The level of CAT enzyme in mice treated with *H. aitchisonii* at the same dosage as animals treated with LPS rose considerably (*p* < 0.001) but was lower than that of mice treated with aspirin (Figure 5D).

### 3.6 Docking studies

**FIGURE 6:**
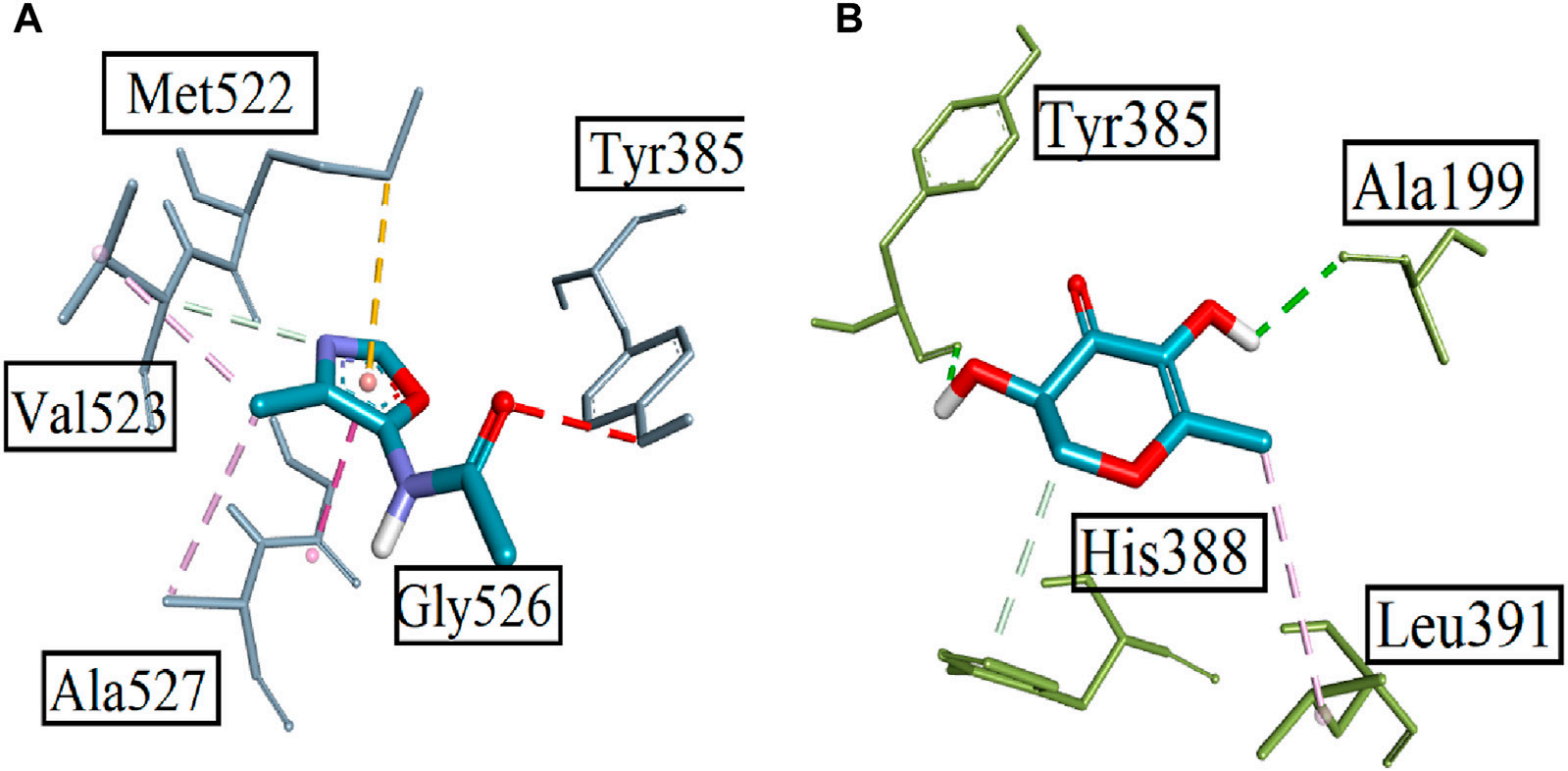
Displaying the 3D visualization of **(A)** Compound **1** and **(B)** Compound **2**, inside the binding pocket of targeted protein PDB ID 1CX2.

Computational studies were performed to analyze the potential of the 18 compounds identified by GC-MS against the targeted COX-2 protein (1CX2) through docking software AutoDock Vina. The validation of the docking protocol was performed through the re-docking method. All the identified compounds were docked into the active site of 1CX2, and analysis of docking potential was carried out through binding energies. The computed binding energies were found to be in the range of −4.258 Kcal/mol to −7.417 Kcal/mol. Compounds 1, 2, 8, and 11 were found to be prominent, with more negative binding energy values than others. These values were −7.417 Kcal/mol, −6.854 Kcal/mol, −6.952 Kcal/mol, and −7.235 Kcal/mol. The binding energies of all the identified molecules with the target protein are given in Supplementary Table S2. When docked with the targeted protein, Compound **1** showed good binding interactions. The prominent interactions were a carbon–hydrogen bond with Val 523 (3.97 Å), a pi–sulfur bond with Met 522 (5.13 Å), and an amide pi-stacked bond (3.84 Å) with Gly 526. Other interactions were found with Tyr 385 and Ala 527. Compound **2** gave two conventional hydrogen bonds with Tyr 385 (2.61 Å) and Ala 199 (2.39 Å), a carbon–hydrogen bond with His 388 (3.65 Å), and an alkyl bond with Leu 391 (4.82 Å). The interactions are shown in Figure 6. Compound 8, when docked with COX protein COX-2 (1CX2), showed conventional hydrogen bonds with Glu 465 (2.59 Å) and Cys 41 (2.45 Å). A carbon–hydrogen bond was found with Glu 45 and Arg 44 at bond lengths of 3.69 Å and 3.62 Å. Compound **11** displayed a conventional hydrogen bond with Lys 137 (2.87 Å) and alkyl interactions with Pro 153, Cys 47, Leu 152, Cys 36, and Arg 469 (Supplementary Figure S2).

## 4 Discussion

Natural products and their phytocomponents are used to treat a wide range of biological problems (Sahu and Sahu, 2015; Sameena and Thoppil, 2022). The pharmacological activities of a plant depend heavily on its phytochemicals (Rizvi et al., 2022). The active medicinal component is seen as a single molecule. It is also evident that many therapeutic plants have many functions (Zafar et al., 2019). The synergistic action of the phytochemicals may account for the wide variety and potency of the pharmacological activity seen in medicinal plants (Jongrungraungchok et al., 2023). One of the primary goals of ethnomedicine and medicinal plants is to reduce pain and inflammation via the pharmacological properties of plants. Analgesia, inflammation, and antioxidant control are all areas in which medicinal herbs have a long history of use. The traditional usage of plants as medicines is becoming recognized and validated in the modern era. We can make a scientific case for the occurrence of these activities if we can identify the individual phytochemicals responsible for them and back up the claims with *in vitro*, *in vivo,* and *in silico* evidence. In this study, we used GC-MS analysis to identify 18 phytocomponents in *H. aitchisonii*. The synergistic action of the discovered phytochemicals may be credited with the accomplished activities (Álvarez-Vásquez et al., 2022; Guzmán-Gutiérrez et al., 2023).

Ethyl alpha-D-galactopyranoside is the most abundant chemical ingredient of *H. aitchisonii*, as measured by peak area (Table 1), and it is also a highly studied biomolecule with purported therapeutic effects. Antiviral (Liao et al., 2013), anti-androgenic (Suphrom et al., 2012), anticancer (Ye et al., 2017), neuroprotective (Wu et al., 2019), and antioxidant (Lei et al., 2019) biological properties have been described. Pentadecanoic acid, 14-methyl-, methyl ester was the second most common chemical by concentration (21.92%), as indicated in Table 1. The extract contains pentadecanoic acid, 14-methyl-ester, which has been shown to block both catecholamine O-methyl transferase and methyl guanidine synthesis. The catecholamines (adrenaline, noradrenaline, and dopamine) are broken down by the enzyme catecholamine O-methyl transferase (COMT). Therefore, a COMT inhibitor prevents the neurotransmitter catecholamines from degrading. In particular, COMT inhibitors may be used to treat Parkinson’s disease because dopamine is a crucial neurotransmitter in the basal ganglia. Methyl guanidine (MG) has been identified as a neurotoxin and a nephrotoxin (Westfall and Westfall, 2011). It is produced from creatinine (CRN) by hydroxyl radicals and other reactive oxygen species (ROS) (Sadiq et al., 2015). Therefore, the antioxidant activity of CRN may be inferred from the compound’s ability to prevent hydroxyl radical-mediated MG production. Because factors like soil type, photoperiod, light intensity, relative humidity, temperature, wind and sun exposure, climate variability, season of the year, plant development, and attitude variation stage can shift the quantitative and qualitative variation of a plant, as well as its biological effects, more research is needed to assess the persuade of environmental circumstances like these (Elmardy et al., 2021).

The development of new medicines is necessary because pain is reported to be the main sign of many diseases, and there is great concern about insufficient pain control despite the existence of analgesic medicines, in addition to the many adverse reactions that these molecules can cause, such as chemical dependency (Volkow and McLellan, 2016). The chemical compounds in natural products like essential oils have been shown in recent studies (Jamshidi-Kia et al., 2018) to block or activate nociceptive receptors, among other mechanisms, making them promising candidates for drug discovery to control pain. The acetic acid-induced writhing test is a common method for quickly gauging the analgesic efficacy of potential medications. It is broadly employed to detect the antinociceptive effects of central nervous system inhibitors like opioids and peripheral nervous system inhibitors like NSAIDs (Barreto et al., 2016). This model of nociception is nonspecific because it causes peripheral sensitization in animals through endogenous nociceptive chemicals that are released indirectly.

Drug-induced inoculation consists of two stages. In the first (neurogenic) phase, substance P is released and works as a neurotransmitter to transport pain signals from nociceptors through C fibers to the dorsal horn of the spinal cord, bypassing the inflammatory process. Pain is formed in the second (inflammatory) phase when histamine, serotonin, bradykinin, and PGs are released in response to inflammation in the tissue wounded by formalin. Peripherally acting medications, such as anti-inflammatories (Mohammadifard and Alimohammadi, 2018), are only efficacious in the second phase of the formalin test, whereas centrally acting pharmaceuticals, such as opioid analgesics, suppress both phases. In a dose-dependent way, we found that the tested samples of *H. aitchisonii* decreased the pain response to both phases of the formalin test, demonstrating an antinociceptive impact at both the central and peripheral levels. This finding suggests that *H. aitchisonii* is not only antinociceptive but also anti-inflammatory.

Histamine, serotonin, and bradykinin are just a few of the proinflammatory markers that are released in response to carrageenan injection, and this is followed by an increase in the activity of COX and nitric oxide synthase (NOS), with the peak of proinflammatory molecule release happening 2 h after the injection. Our findings suggested that *H. aitchisonii* inhibited edema formation for 5 consecutive hours despite the fact that the drug molecule normally requires a protein structure as a target for binding for a specific pharmacological activity, implying that the molecules from essential oil can inhibit more than one cellular signaling pathway (Mansouri et al., 2015). To obtain the 3D structure of the target protein, the molecular docking studies employ specialized software and available protein data banks (PDBs). Possible interactions between compounds/phytochemicals and the target protein are determined by the binding energies of the compounds/phytochemicals and the protein (Kashyap et al., 2015). All the binding energies of the phytochemicals with the target protein were calculated using a molecular docking technique. Eighteen compounds were docked in this research project, and the docking binding energies of the molecules were linked and compared. We show a small subset of compounds based on their binding energies in Figure 5. These phytochemicals have a synergistic interaction with the target protein is supported by the docking results.

## 5 Conclusion

The results showed that the traditional usage of *H. aitchisonii* to treat pain, inflammation, and a wide variety of skin illnesses is supported by its ethnopharmacological qualities. Acute toxicity tests on *H. aitchisonii* showed that it was quite safe in acute exposure, with no fatality occurring until a dose of 3,000 mg/kg. Therefore, it is concluded that this plant is a source of secondary metabolites with applications in pain, inflammation, and antioxidant therapy.

## Data Availability

The original contributions presented in the study are included in the article/Supplementary Material; further inquiries can be directed to the corresponding authors.
