# Supplementary material for: Evaluation of Habenaria aitchisonii Reichb. for antioxidant, anti-inflammatory, and antinociceptive effects with in vivo and in silico approaches
Source: Front Chem. 2024 Mar 19;12:1351827. doi: 10.3389/fchem.2024.1351827 (PMC10985259; doi:10.3389/fchem.2024.1351827)
Supplement: Supplementary file 1 [file DataSheet1.doc]

Type of the Paper (Article)

GCMS analysis, antioxidant, anti-inflammatory, antinociceptive and Insilco approach of *Habenaria aitchisonii* reichb.

Saeed Ahmed Asiri^1^, Madeeha Shabnam^2^, Rehman Zafar^3^, Osama M.Alshehri^4^, Mohammed Ali alshehri^1^, Abdul Sadiq^5^, Mater H. Mahnashi^6^*, Muhammad Saeed Jan^7*^

^1^Department of Clinical Laboratory Sciences, Faculty of Applied Medical Sciences, Najran University, 1988, Najran, 61441, Saudi Arabia. ([saaasiri@nu.edu.sa](mailto:saaasiri@nu.edu.sa); [maab515@nu.edu.sa](mailto:maab515@nu.edu.sa))

^2^Department of Chemistry, Women University, Mardan, KP, Pakistan ([shabnammadeeha4@gmail.com](mailto:shabnammadeeha4@gmail.com))

^3^Akhtar Saeed College of Pharmacy, Bahria Golf City, Murree Expressway, Rawalpindi ([rehman.zafar@amdc.edu.pk](mailto:rehman.zafar@amdc.edu.pk))

^4^Department of Clinical Laboratory Sciences, College of Applied Medical Sciences, Najran University, Najran, Saudi Arabia ([Omalshehri@nu.edu.sa](mailto:Omalshehri@nu.edu.sa))

^5^Department of Pharmacy, Faculty of Biological Sciences, University of Malakand, Chakdara, 18000 Dir (L), KP, Pakistan ([sadiquom@yahoo.com](mailto:sadiquom@yahoo.com))

^6^Department of pharmaceutical chemistry, college of pharmacy, Najran University, Najran, Kingdom of Saudi Arabia ([mhmahneshi@nu.edu.sa](mailto:mhmahneshi@nu.edu.sa))

^7^Department of Pharmacy, Bacha Khan University, Charsadda 24420, KP, Pakistan ([saeedjan@bkuc.edu.pk](mailto:saeedjan@bkuc.edu.pk))

*** Correspondence should be addressed to** **Muhammad Saeed Jan;** Bacha Khan University Charsadda, KP, Pakistan **Email:** [saeedjan@bkuc.edu.pk](mailto:saeedjan@bkuc.edu.pk); Cell #: 0092 315 3109610 **& Mater H. Mahnashi**([mhmahneshi@nu.edu.sa](mailto:mhmahneshi@nu.edu.sa))

**Table S1: The GC-MS detail of the identified compounds.**

| **S. No.** | **Name of the Compound** | **R. Time (min.)** | **Area** | **Conc. (%)** | **Formula** |
| --- | --- | --- | --- | --- | --- |
|  | N-[(4-Methyl-1,3-oxazol-5yl)carbonyl]acetamide | 5.571 | 782699 | 7.81 | C_6_H_8_N_2_O_2_ |
|  | 3,5-Dihydroxy-6-methyl-2,3-dihydro-4H-pyran-4-one | 6.966 | 638333 | 6.37 | C_6_H_8_O_4_ |
|  | Phenol, 2,6-dimethoxy- | 11.832 | 63328 | 0.63 | C_8_H_10_O_3_ |
|  | 2,7-Octadiene-1,6-diol, 2,6-dimethyl- | 12.099 | 169201 | 1.69 | C_10_H_18_O_2_ |
|  | Propanenitrile, 3,3-dimethoxy- | 12.404 | 296113 | 2.95 | C_5_H_9_NO_2_ |
|  | 3’ ,5’-Dimethoxyacetophenone | 16.983 | 182954 | 1.82 | C_10_H_12_O_3_ |
|  | Nonanedioic acid, dimethyl ester | 17.477 | 60599 | 0.60 | C_11_H_20_O_4_ |
|  | Methyl alpha-D-galactopyranoside | 18.133 | 2693910 | 26.87 | C_7_H_14_O_6_ |
|  | 4-((1E)-3-Hydroxy-1-propenyl)-2-methoxyphenol | 20.886 | 325096 | 3.24 | C_10_H_12_O_3_ |
|  | 7-Metyl-Z-tetradecen-1-enyl acetate | 21.803 | 314143 | 3.13 | C_17_H_32_O_2_ |
|  | Pentadecanoic acid, 14-methyl-, methyl ester | 24.746 | 2197445 | 21.92 | C_17_H_34_O_2_ |
|  | n-Hexadecanoic aid | 25.441 | 328782 | 3.28 | C_16_H_32_O_2_ |
|  | Hexadecanoic acid, ethyl ester | 26.076 | 44529 | 0.44 | C_18_H_36_O_2_ |
|  | 9, 12-Octadecanoic acid, methyl ester | 27.972 | 535152 | 5.34 | C_19_H_34_O_2_ |
|  | 9,12-Octadedcadienoyl chloride, (Z,Z)- | 28.093 | 894944 | 8.93 | C_18_H_31_ClO |
|  | Phytol | 28.341 | 95714 | 0.95 | C_20_H_42_O |
|  | Octadecanoic acid, methyl ester | 28.582 | 316918 | 3.16 | C_19_H_38_O_2_ |
|  | 7-Tetradecenal, (Z)- | 28.805 | 86998 | 0.87 | C_14_H_26_O |

**Table S2: Binding energy values in Kcal/mol of the identified compounds via computational studies**

| **S.No.** | **Name of the Compound** | **Binding Energies**  **(Kcal/mol)** |
| --- | --- | --- |
|  | N-[(4-Methyl-1,3-oxazol-5yl)carbonyl]acetamide | -7.417 |
|  | 3,5-Dihydroxy-6-methyl-2,3-dihydro-4H-pyran-4-one | -6.854 |
|  | Phenol, 2,6-dimethoxy- | -6.321 |
|  | 2,7-Octadiene-1,6-diol, 2,6-dimethyl- | -4.982 |
|  | Propanenitrile, 3,3-dimethoxy- | -6.548 |
|  | 3’ ,5’-Dimethoxyacetophenone | -5.256 |
|  | Nonanedioic acid, dimethyl ester | -5.478 |
|  | Methyl alpha-D-galactopyranoside | -6.952 |
|  | 4-((1E)-3-Hydroxy-1-propenyl)-2-methoxyphenol | -4.365 |
|  | 7-Metyl-Z-tetradecen-1-enyl acetate | -4.875 |
|  | Pentadecanoic acid, 14-methyl-, methyl ester | -7.235 |
|  | n-Hexadecanoic aid | -4.258 |
|  | Hexadecanoic acid, ethyl ester | -5.987 |
|  | 9, 12-Octadecanoic acid, methyl ester | -5.847 |
|  | 9,12-Octadedcadienoyl chloride, (Z,Z)- | -5.235 |
|  | Phytol | -6.002 |
|  | Octadecanoic acid, methyl ester | -6.854 |
|  | 7-Tetradecenal, (Z)- | -5.023 |

**
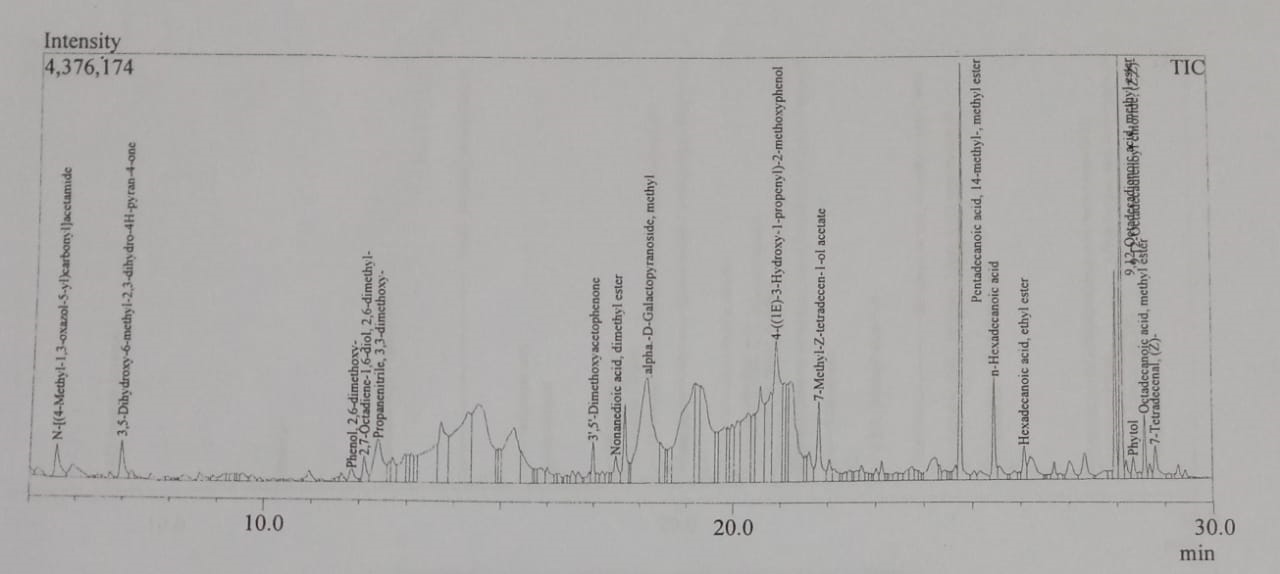
**

**Figure S1:** GCMS spectra of *Habenaria aitchisonii.*


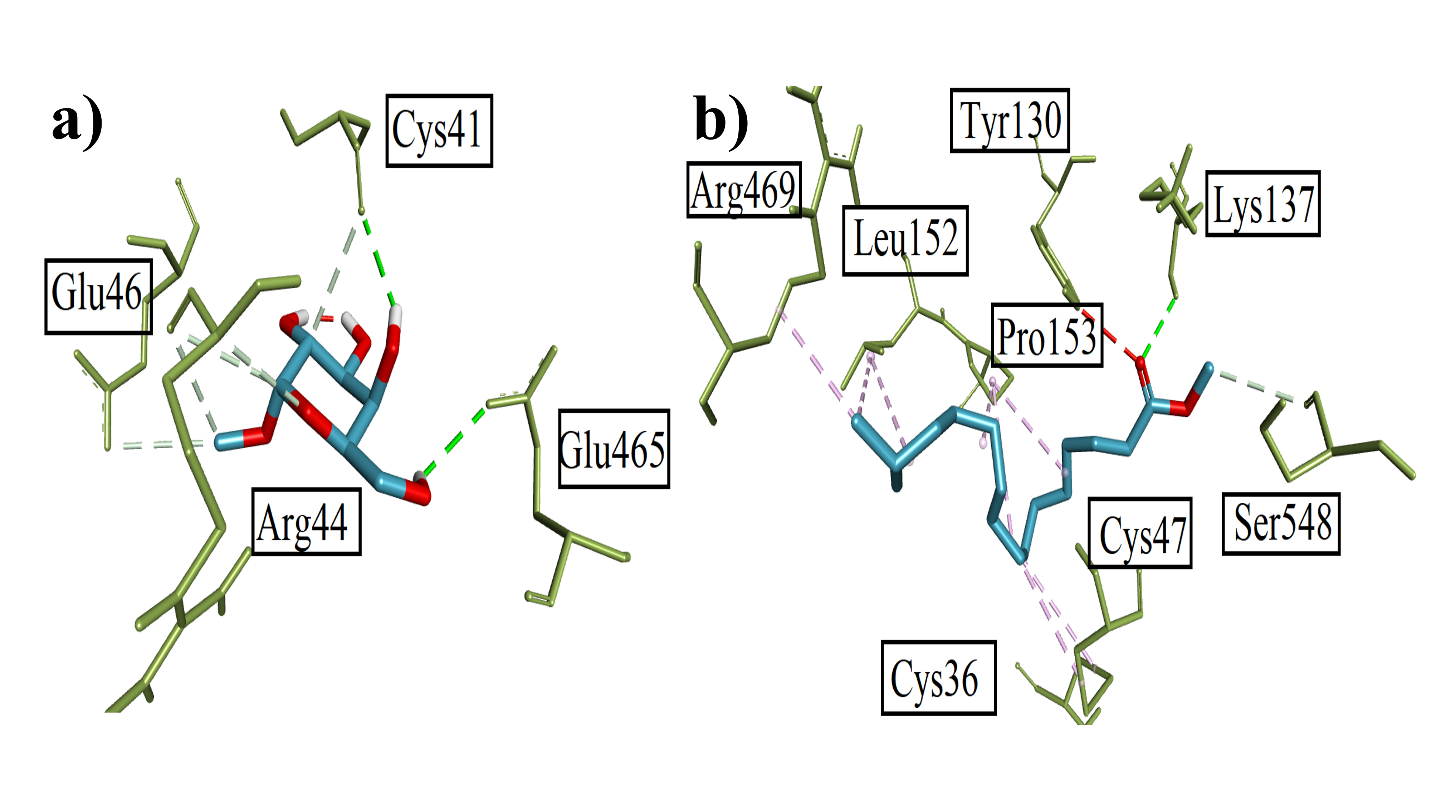


**Figure S2:** Displaying the 3D visualization of **a)** Compound 8,**b)** Compound 11, inside the binding pocket of targeted protein pdb Id 1CX2.
